# Supplementary material for: Long-Term Effects on Gonadal Function After Treatment of Colorectal Cancer: A Systematic Review and Meta-Analysis
Source: Cancers (Basel). 2024 Nov 29;16(23):4005. doi: 10.3390/cancers16234005 (PMC11640400; doi:10.3390/cancers16234005)
Supplement: Supplementary file 1 [file cancers-16-04005-s001.zip › cancers-3275707-supplementary.pdf]

44Search Strategies

Detailed search strategy

To identify potentially relevant publications on the topic, a search strategy was designed, and investigated in MEDLINE, Embase, and Cochrane Library. A medical information specialist developed an initial search strategy in Embase and tested it against a list of core references to ensure key publications were included. After refinement, the information specialist set up the search strategy for each information source based on database-specific index terms and free text. The free text search included synonyms, acronyms, and similar terms. In all databases the in-built publication year filter was applied to limit the results for the period from 2000 to present. Additionally, in Cochrane the results were filtered by trials and systematic reviews. No other database-provided limits have been applied in any sources considering study types, languages or any other formal criteria. Studies concerning exclusively animals were excluded from the searches by using a double-negative search strategy based on the "Humans only" filters by Ovid. The search was finalized on 07/03/2024. The results were deduplicated using the automated deduplication tool Deduklick (https://www.risklick.ch/products/deduklick/)[1]. The Covidence screening tool has detected and removed a further 11 duplicates.

Search date	Database searched	Platform	Years of coverage	Records
7 March 2024	Embase	Ovid	1974 - 2024 March 06	3381
7 March 2024	Medline	Ovid	1946 - 2024 March 06	949
7 March 2024	Cochrane Database of Systematic Reviews	Wiley	1992 - present	2
7 March 2024	Cochrane Central Register of Controlled Trials	Wiley	1992 - present	88
			Sum of references	4420
			Removed duplicates	- 839
			**Total references**	**3581**

****

Search strategy used per database

Embase <1974 to 2024 March 06>

1 (((Colorect* or colo-rect* or rectum or colon or colonic or rectal or recto-colonic or rectocolonic or rectosigmoid or recto-sigmoid or colitis) adj4 (Neoplas* or tumor* or tumour* or cancer* or malignanc* or cacino* or metastas*)) or crc).ti,ab. 382951

2 exp colorectal cancer/ 398205

3 or/1-2 499884

4 (((therap* or intervention*) adj3 (biologic* response modifier* or brm or immun* or mesenchymal stromal cell or antibod* or mab*)) or (photoimmunotherap* or radioimmunotherapy* or cryoimmunotherap* or immunotherapy*) or (inhibit* adj2 (kinase or multi-kinase or small

[1] Borissov N, Haas Q, Minder B, et al. Reducing systematic review burden using Deduklick: a novel, automated, reliable, and explainable deduplication algorithm to foster medical research. Syst Rev. 2022;11(1):172. Published 2022 Aug 17. doi:10.1186/s13643-022-02045-9

molecule* or proteasome or heat shock protein or epidermal growth factor receptor or EGFR or Vascular endothelial growth factor or VEGF)) or (anti-EGFR or anti-VEGF) or (promot* adj2 apoptos*) or (Imatinib or Gleevec or Glivec or Pazopanib or Pazovas or Pazopex or Pazomax or Pazolon or Pazopure or Pazopen or Pazoblast or Pazosin or Pazolife or Pazocure or Lapatinib or Tykerb or Bevacizumab or Avastin or Cetuximab or Erbitux or Panitumumab or Vectibix or Vasculotropin or CTP37-DT or Lenvatinib or Lenvima or Olaratumab or Lartruvo or Ramucirumab or Cyramza or Fluoropyrimidine* or 5-fluorouracil or 5-FU or capecitabine or TAS102 or FOLFOX or FOLFOXIRI or Regorafenib or encorafenib or binimetinib or oxaliplatin)).ti,ab. 726386

5 exp immunotherapy/ or exp protein kinase inhibitor/ 997492

6 (chemotherap* or chemo-therap* or chemoradiotherap* or chemo-radiotherap* or adjuvant drug therap* or carcinochemotherap* carcino-chemotherap* or antineoplastic agent* or anti-neoplastic agent* or antineoplastic drug* or anti-neoplastic drug* or antitumo?r agent* or anti-tumo?r agent* or antitumo?r drug* or anti-tumo?r drug* or anticancer* agent* or anti-cancer* agent* or anticancer* drug* or anti-cancer drug* or anticarcinogen* or anti-carcinogen* or anticancerogen* or anti-cancerogen* or ((cancer* or tumo?r* or neoplas*) adj3 treat*)).ti,ab. 1333575

7 exp antineoplastic agent/ or exp multimodality cancer therapy/ or exp cancer chemotherapy/ or exp antineoplastic protocol/ 3211383

8 (Radio-therap* or radiotherap* or radiationtherap* or chemoradiotherap* or radiochemotherap* or protontherap* or radiosurg* or radio-surg* or irradiation* or x-ray-therap* or therap* radiolog* or IMRT* or IORT* or radioimmunotherap* or radio-immuno-therap* or ((radiat* or irradiat* or radioisotope* or radio-isotope* or chemoradio or chemo-radio or radiochemo or radio-chemo or proton or x-ray or xray) adj2 (therap* or oncolog* or brachytherap* or brachy-therap*))).ti,ab. 723691

9 exp cancer radiotherapy/ 342222

10 or/4-9 4642920

11 (fertili#ation* or fertility or fertile or fecund* or subfecund* or sub-fecund* or infecund* or infertility or (ovar* adj4 sterility) or subfert* or sub-fert* or anovularit* or gonad* or reproductive organ* or reproduction* or gamete-producing gland* or ovarian reserve* or ovary or ovaries or ovarian follicle* or oogenes#s or oocyte* or amenorrhea* or premature menopaus* or early menopaus* or climacterium pr?ecox or Gonadotropin* or AMH or Anti-Mu?llerian Hormone* or Antimu?llerian Hormone* or Anti-Mu?llerian Factor* or Mu?llerian Inhibiting Hormone* or mu?llerian inhibitor* or FSH or Follicle Stimulating Hormone* or Folliculostimulating Hormone* or Follitropin or FSH-releasing hormone* or LH-FSH or testis or testes or testicle* or spermatogenes* or sperm* or semen or gametogenes* or hypogonadism* or hypo-gonadism* or "reproductive system*" or azoospermia* or spermatozoon* or ((resumpt* or recover*) adj3 menstruat*) or menarch* or "menstruat* onset*" or "first menstruat*" or "puberal h?emorrhage*" or (?estrogen* adj3 (substitut* or administrat* or replace* or therap*)) or "hormon* replacement therap*" or hrt or inhibin b or inhibin subunit b or (follicular matur* or ovul* or oligomenorrhoe* or folliculo* or oncofertil*)).ti,ab. 941823

12 exp fertility/ or exp infertility/ or exp semen analysis/ or exp gonad/ or exp amenorrhea/ or exp early menopause/ or reproduction/ or gametogenesis/ or spermatozoon/ or exp menarche/ or exp estrogen therapy/ or exp inhibin B/ or exp testis size/ 624420

13 or/11-12 1107140

14 3 and 10 and 133876

15 (exp animal/ or exp invertebrate/ or nonhuman/ or animal experiment/ or animal tissue/ or animal model/ or exp plant/ or exp fungus/ or (rat? or mouse or mice or animal?).ti.) not (exp human/ or human tissue/) 7917741

16 14 not 15 3645

17 limit 16 to yr="2000 -Current" 3381

****

Ovid MEDLINE(R) ALL <1946 to March 06, 2024>

1 (((Colorect* or colo-rect* or rectum or colon or colonic or rectal or recto-colonic or rectocolonic or rectosigmoid or recto-sigmoid or colitis) adj4 (Neoplas* or tumor* or tumour* or cancer* or malignanc* or cacino* or metastas*)) or crc).ti,ab. 257955

2 exp Colorectal Neoplasms/ 243894

3 or/1-2 330704

4 (((therap* or intervention*) adj3 (biologic* response modifier* or brm or immun* or mesenchymal stromal cell or antibod* or mab*)) or (photoimmunotherap* or radioimmunotherapy* or cryoimmunotherap* or immunotherapy*) or (inhibit* adj2 (kinase or multi-kinase or small molecule* or proteasome or heat shock protein or epidermal growth factor receptor or EGFR or Vascular endothelial growth factor or VEGF)) or (anti-EGFR or anti-VEGF) or (promot* adj2 apoptos*) or (Imatinib or Gleevec or Glivec or Pazopanib or Pazovas or Pazopex or Pazomax or Pazolon or Pazopure or Pazopen or Pazoblast or Pazosin or Pazolife or Pazocure or Lapatinib or Tykerb or Bevacizumab or Avastin or Cetuximab or Erbitux or Panitumumab or Vectibix or Vasculotropin or CTP37-DT or Lenvatinib or Lenvima or Olaratumab or Lartruvo or Ramucirumab or Cyramza or Fluoropyrimidine* or 5-fluorouracil or 5-FU or capecitabine or TAS102 or FOLFOX or FOLFOXIRI or Regorafenib or encorafenib or binimetinib or oxaliplatin)).ti,ab. 477743

5 exp Immunotherapy/ 342294

6 exp Protein Kinase Inhibitors/ 124328

7 (chemotherap* or chemo-therap* or chemoradiotherap* or chemo-radiotherap* or adjuvant drug therap* or carcinochemotherap* carcino-chemotherap* or antineoplastic agent* or anti-neoplastic agent* or antineoplastic drug* or anti-neoplastic drug* or antitumo?r agent* or anti-tumo?r agent* or antitumo?r drug* or anti-tumo?r drug* or anticancer* agent* or anti-cancer* agent* or anticancer* drug* or anti-cancer drug* or anticarcinogen* or anti-carcinogen* or anticancerogen* or anti-cancerogen* or ((cancer* or tumo?r* or neoplas*) adj3 treat*)).ti,ab. 886286

8 exp Antineoplastic Agents/ or exp Combined Modality Therapy/ or exp Antineoplastic Combined Chemotherapy Protocols/ or exp chemotherapy, adjuvant/ or exp Antineoplastic Protocols/ or exp chemoradiotherapy/ or radioimmunotherapy/ or exp radiotherapy, adjuvant/ 1514256

9 (Radio-therap* or radiotherap* or radiationtherap* or chemoradiotherap* or radiochemotherap* or protontherap* or radiosurg* or radio-surg* or irradiation* or x-ray-therap* or therap* radiolog* or IMRT* or IORT* or radioimmunotherap* or radio-immuno-therap* or ((radiat*

or irradiat* or radioisotope* or radio-isotope* or chemoradio or chemo-radio or radiochemo or radio-chemo or proton or x-ray or xray) adj2 (therap* or oncolog* or brachytherap* or brachy-therap*))).ti,ab. 521704

10 exp Radiotherapy/ 210370

11 or/4-10 2863097

12 (fertili#ation* or fertility or fertile or fecund* or subfecund* or sub-fecund* or infecund* or infertility or (ovar* adj4 sterility) or subfert* or sub-fert* or anovularit* or gonad* or reproductive organ* or reproduction* or gamete-producing gland* or ovarian reserve* or ovary or ovaries or ovarian follicle* or oogenesis or oocyte* or amenorrhea* or premature menopaus* or early menopaus* or climacterium pr?ecox or Gonadotropin* or AMH or Anti-Mu?llerian Hormone* or Antimu?llerian Hormone* or Anti-Mu?llerian Factor* or Mu?llerian Inhibiting Hormone* or mu?llerian inhibitor* or FSH or Follicle Stimulating Hormone* or Folliculostimulating Hormone* or Follitropin or FSH-releasing hormone* or LH-FSH or testis or testes or testicle* or spermatogenes* or sperm* or semen or gametogenes* or hypogonadism* or hypo-gonadism* or "reproductive system*" or azoospermia* or spermatozoon* or ((resumpt* or recover*) adj3 menstruat*) or menarch* or "menstruat* onset*" or "first menstruat*" or "puberal h?emorrhage*" or (?estrogen* adj3 (substitut* or administrat* or replace* or therap*)) or "hormon* replacement therap*" or hrt or inhibin b or inhibin subunit b).ti,ab. 776983

13 exp Fertility/ or exp Infertility/ or exp Gonads/ or Amenorrhea/ or anovulation/ or menopause, premature/ or Reproduction/ or Gametogenesis/ or Spermatozoa/ or exp Menarche/ or exp Estrogen Replacement Therapy/ or exp Inhibin-beta Subunits/ 402991

14 or/12-13 894649

15 3 and 11 and 141259

16 (exp animal/ or exp invertebrate/ or animal experiment/ or animal model/ or exp plant/ or exp fungus/ or (rat? or mouse or mice or animal?).ti.) not exp human/ 5785489

17 15 not 16 1184

18 limit 17 to yr="2000 -Current" 949

****

Cochrane Database of Systematic Reviews and Central Register of Controlled Trials

#1 (((Colorect* or colo-rect* or rectum or colon or colonic or rectal or recto-colonic or rectocolonic or rectosigmoid or recto-sigmoid or colitis) NEAR/4 (Neoplas* or tumor* or tumour* or cancer* or malignanc* or cacino* or metastas*)):ti,ab or crc):ti,ab 24464

#2 [mh "Colorectal Neoplasms"] 12601

#3 #1 OR #2 26732

#4 ((therap* OR intervention*) NEAR/3 ((biologic* NEXT response NEXT modifier*) OR brm OR immun* OR (mesenchymal NEXT stromal NEXT cell) OR antibod* OR mab*)):ti,ab OR ((therap* OR intervention*) NEAR/3 ((biologic* NEXT response NEXT modifier*) OR brm OR immun* OR (mesenchymal NEXT stromal NEXT cell) OR antibod* OR mab*)):ti,ab OR (photoimmunotherap* OR radioimmunotherapy* OR cryoimmunotherap* OR immunotherapy*):ti,ab OR (inhibit* NEAR/2

(kinase OR multi-kinase OR (small NEXT molecule*) OR proteasome OR (heat NEXT shock NEXT protein) OR (epidermal NEXT growth NEXT factor NEXT receptor) OR egfr OR (vascular NEXT endothelial NEXT growth NEXT factor) OR vegf)):ti,ab OR ((anti NEXT egfr) OR (anti NEXT vegf) OR (promot* NEAR/2 apoptos*) OR Imatinib OR Gleevec OR Glivec OR Pazopanib OR Pazovas OR Pazopex OR Pazomax OR Pazolon OR Pazopure OR Pazopen OR Pazoblast OR Pazosin OR Pazolife OR Pazocure OR Lapatinib OR Tykerb OR Bevacizumab OR Avastin OR Cetuximab OR Erbitux OR Panitumumab OR Vectibix OR Vasculotropin OR (ctp37 NEXT dt) OR Lenvatinib OR Lenvima OR Olaratumab OR Lartruvo OR Ramucirumab OR Cyramza OR Fluoropyrimidine* OR (5 NEXT fluorouracil) OR (5 NEXT fu) OR capecitabine OR tas102 OR folfox OR folfoxiri OR Regorafenib OR encorafenib OR binimetinib OR oxaliplatin):ti,ab 51534

#5 [mh Immunotherapy] 12047

#6 (chemotherap* or chemo-therap* or chemoradiotherap* or chemo-radiotherap* or (adjuvant NEXT drug NEXT therap*) or carcinochemotherap* carcino-chemotherap* or (antineoplastic NEXT agent*) or (anti-neoplastic NEXT agent*) or (antineoplastic NEXT drug*) or (anti-neoplastic NEXT drug*) or (antitumor NEXT agent*) or (antitumour NEXT agent*) or (anti-tumor NEXT agent*) or (anti-tumour NEXT agent*) or (antitumor NEXT drug*) or (antitumour NEXT drug*) or (anti-tumor NEXT drug*) or (anti-tumour NEXT drug*) or (anticancer* NEXT agent*) or (anti-cancer* NEXT agent*) or (anticancer* NEXT drug*) or (anti-cancer NEXT drug*) or anticarcinogen* or anti-carcinogen* or anticancerogen* or anti-cancerogen* or ((cancer* or tumor* or tumour*or neoplas*) NEAR/3 treat*)):ti,ab,kw 124882

#7 [mh "Antineoplastic Agents"] OR [mh "Combined Modality Therapy"] OR [mh "Antineoplastic Combined Chemotherapy Protocols"] OR [mh "chemotherapy, adjuvant"] OR [mh "Antineoplastic Protocols"] OR [mh "Chemoradiotherapy"] OR [mh ^Radioimmunotherapy] OR [mh "Radiotherapy, Adjuvant"] 58367

#8 (Radio-therap* or radiotherap* or radiationtherap* or chemoradiotherap* or radiochemotherap* or protontherap* or radiosurg* or radio-surg* or irradiation* or x-ray-therap* or (therap* NEXT radiolog*) or IMRT? or IORT? or radioimmunotherap* or radio-immuno-therap* or ((radiat* or irradiat* or radioisotope* or radio-isotope* or chemoradio or chemo-radio or radiochemo or radio-chemo or proton or x-ray or xray) NEAR/2 (therap* or oncolog* or brachytherap* or brachy-therap*))):ti,ab,kw 53293

#9 [mh Radiotherapy] 9984

#10 #4 OR #5 OR #6 OR #7 OR #8 OR #9 198445

#11 (fertili*ation* OR fertility OR fertile OR fecund* OR subfecund* OR sub-fecund* OR infecund* OR infertility OR (ovar* NEAR/4 sterility) OR subfert* OR sub-fert* OR anovularit* OR gonad* OR (reproductive NEXT organ*) OR reproduction* OR (gamete-producing NEXT gland*) OR (ovarian NEXT reserve*) OR ovary OR ovaries OR (ovarian NEXT follicle*) OR oogenesis OR oocyte* OR amenorrhea* OR (premature NEXT menopaus*) OR (early NEXT menopaus*) OR (climacterium NEXT pr*ecox) OR Gonadotropin* OR AMH OR (Anti-Mu*llerian NEXT Hormone*) OR (Antimu*llerian NEXT Hormone*) OR (Anti-Mu*llerian NEXT Factor*) OR (Mu*llerian NEXT Inhibiting NEXT Hormone*) OR (mu*llerian NEXT inhibitor*) OR FSH OR (Follicle NEXT Stimulating NEXT Hormone*) OR (Folliculostimulating NEXT hormone*) OR Follitropin OR (FSH-releasing hormone*) OR LH-FSH OR testis OR testes OR testicle* OR spermatogenes* OR sperm* OR semen OR gametogenes* OR hypogonadism* OR hypo-gonadism* OR (reproductive NEXT system*) OR azoospermia* OR spermatozoon* OR ((resumpt* OR recover*) NEAR/3 menstruat*) OR menarch* OR (menstruat* NEXT onset*) OR (first NEXT menstruat*) OR (puberal NEXT h*emorrhage*) OR (*estrogen* NEAR/3

(substitut* OR administrat* OR replace* OR therap*)) OR (hormon* NEXT replacement NEXT therap*) OR hrt OR (inhibin NEXT b) OR (inhibin NEXT subunit NEXT b)):ti,ab 41563

#12 [mh Fertility] OR [mh Infertility] OR [mh Gonads] OR [mh ^Amenorrhea] OR [mh ^anovulation] OR [mh ^"menopause, premature"] OR [mh ^Reproduction] OR [mh ^Gametogenesis] OR [mh ^Spermatozoa] OR [mh Menarche] OR [mh "Estrogen Replacement Therapy"] OR [mh "Inhibin-beta Subunits"]9919

#13 #11 OR #12 43534

#14 #3 AND #10 AND #13 96

#15 ([mh animal] OR [mh invertebrate] OR [mh ^"animal experiment"] OR [mh ^"animal model"] OR [mh plant] OR [mh fungus] OR (rat?:ti OR mouse:ti OR mice:ti OR animal?:ti)) NOT [mh human] 13459

#16 #14 NOT #15 with Cochrane Library publication date between Jan 2000 and Mar 2024, in Cochrane Reviews, Trials 90
